# The Erythropoietin/Erythropoietin Receptor Signaling Pathway Promotes Growth and Invasion Abilities in Human Renal Carcinoma Cells

**DOI:** 10.1371/journal.pone.0045122

**Published:** 2012-09-18

**Authors:** Pengjie Wu, Ning Zhang, Xi Wang, Chi Zhang, Teng Li, Xianghui Ning, Kan Gong

**Affiliations:** 1 Department of Urology, Peking University First Hospital, Institute of Urology, Peking University, National Urological Cancer Center, Beijing, People’s Republic of China; 2 Department of Urology, Beijing Chaoyang Hospital, Capital University of Medicine Science, Beijing, People’s Republic of China; 3 Department of General Surgery, Peking University First Hospital, Beijing, People’s Republic of China; University of Central Florida, United States of America

## Abstract

Co-expression of erythropoietin (Epo) and erythropoietin receptor (EpoR) has been found in various non-hematopoietic cancers including hereditary and sporadic renal cell carcinomas (RCC), but the Epo/EpoR autocrine and paracrine mechanisms in tumor progression have not yet been identified. In this study, we used RNA interference method to down-regulate EpoR to investigate the function of Epo/EpoR pathway in human RCC cells. Epo and EpoR co-expressed in primary renal cancer cells and 6 human RCC cell lines. EpoR signaling was constitutionally phosphorylated in primary renal cancer cells, 786-0 and Caki-1 cells, and recombinant human Epo (rhEpo) stimulation had no significant effects on further phosphorylation of EpoR pathway, proliferation, and invasiveness of the cells. Down-regulation of EpoR expression in 786-0 cells by lentivirus-introduced siRNA resulted in inhibition of growth and invasiveness *in vitro* and *in vivo*, and promotion of cell apoptosis. In addition, rhEpo stimulation slightly antagonized the anti-tumor effect of Sunitinib on 786-0 cells. Sunitinib could induce more apoptotic cells in 786-0 cells with knockdown EpoR expression. Our results suggested that Epo/EpoR pathway was involved in cell growth, invasion, survival, and sensitivity to the multi-kinases inhibitor Sunitinib in RCC cells.

## Introduction

Von Hippel-Lindau (VHL) disease is an autosomal dominant inheritable disorder characterized by the inactivation of VHL tumor suppressor gene due to mutations in *VHL* gene [1]–[3]. VHL protein is a component of the ubiquitin ligase E3 protein complex that is involved in the ubiquitination and degradation of hypoxia-inducible factor (HIF) [4], [5]. Loss of VHL function due to *VHL* gene mutation results in the accumulation of HIF, which then induces the over-expression of hypoxia related genes including erythropoietin (Epo) [6], vascular endothelial growth factor (VEGF) [7], [8] and platelet-derived growth factor (PDGF) [9], thus promoting angiogenesis, proliferation and tumorigenesis in multiple organs such as hemangioblastoma in brain and retina, renal cell carcinoma and cyst, pheochromocytoma, pancreatic cyst and tumor.

It becomes clear now that the co-expression of Epo and erythropoietin receptor (EpoR) is not restricted to hematopoietic cells and can be detected in several tumors including RCC [10]–[13]. Epo/EpoR pathway may play more important roles in RCC than in other cancers, because of the specific site of RCC which closely adjoins the Epo secreting tissue [14]. In RCC samples, cytoplasmic Epo and Epo transcript have been frequently found, and the expression of Epo in RCC may be adversely associated with overall survival of the patients [12], [15]–[17]. EpoR can be detected in most RCC tissues and RCC cell lines by immunochemistry, western blotting and reverse transcription-PCR methods [10], [12]–[13]. Epo and EpoR are almost always found in multiple tumors related to VHL disease in brain [18]–[20], retina [21]–[22], pheochromocytoma [23], and endolymphatic sac tumors [24]. In non-hematopoietic tissues, Epo may function as a pleiotropic survival and growth factor. The co-expression of Epo and EpoR in RCC may suggest the autocrine and paracrine mechanisms leading to tumorigenesis and progression of RCC. Functional experiments found that ^125^I-Epo bound to Caki-2 human RCC cell line with intermediate affinity, and Epo induced the increase of cell number dose-dependently in Caki-2 and 786-0 human RCC cell lines and RAG murine RCC cell line [10]. However, the role of Epo/EpoR pathway on promoting RCC progression has not been established.

To evaluate the function of Epo/EpoR pathway in RCC cells, we used RNA interference method to down-regulate EpoR expression in 786-0 cells and then observed the changes in growth, invasiveness, apoptosis, and sensitivity to the clinically used multi-kinases inhibitor Sunitinib. Our data provided the evidences that Epo/EpoR system in RCC may be involved in tumor growth, invasion, survival and sensitivity to Sunitinib.

## Results

### Co-expression of Epo/EpoR in 6 RCC Cell Lines, Primary Renal Tumor Cells and HK-2 Cells

**Figure 1:**
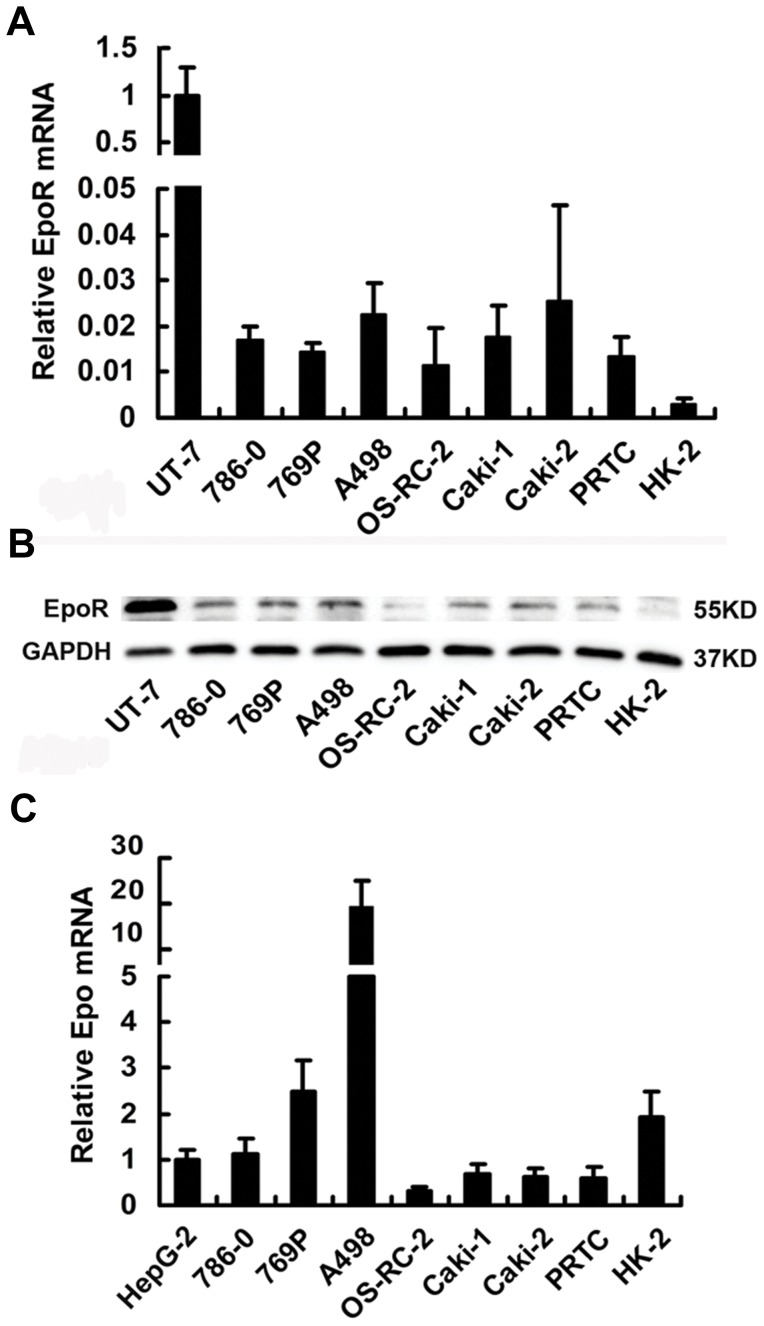
Epo and EpoR co-expression in RCC cell lines and primary renal tumor cells (PRTC) using HK-2, HepG-2 and UT-7 cells as controls. Real-time RT-PCR was used for quantification of (A) EpoR mRNA and (C) Epo mRNA, western blot for (B) EpoR protein detection. Error bar indicates mean ± SD from 3 independent experiments.

RCC cell lines, primary renal tumor cells and the normal renal proximal tubular epithelial cell line HK-2 expressed EpoR mRNA and EpoR protein. RCC cell lines and primary renal tumor cells expressed EpoR higher than HK-2 cells, but much lower than the Epo-dependent UT-7 leukemia cells (Figure 1A and 1B). The specificity of the anti-EpoR antibody was confirmed by the lower EpoR protein expression in 786-0 cells transfected with the EpoR siRNA lentivirus. Compared with the well-defined Epo-expressing HepG-2 cells, all RCC cells and HK-2 expressed Epo mRNA (Figure 1C). However, quantification results of secreted Epo in RCC cells, primary renal tumor cells and HK-2 cultured media were all below the minimal sensitivity limit of the Epo ELISA kit (data not shown). These results are consistent with our previous findings in clinical RCC samples [13].

### Responses of EpoR Pathway, Proliferation Rate and Invasion Ability to rhEpo in RCC Cells

**Figure 2:**
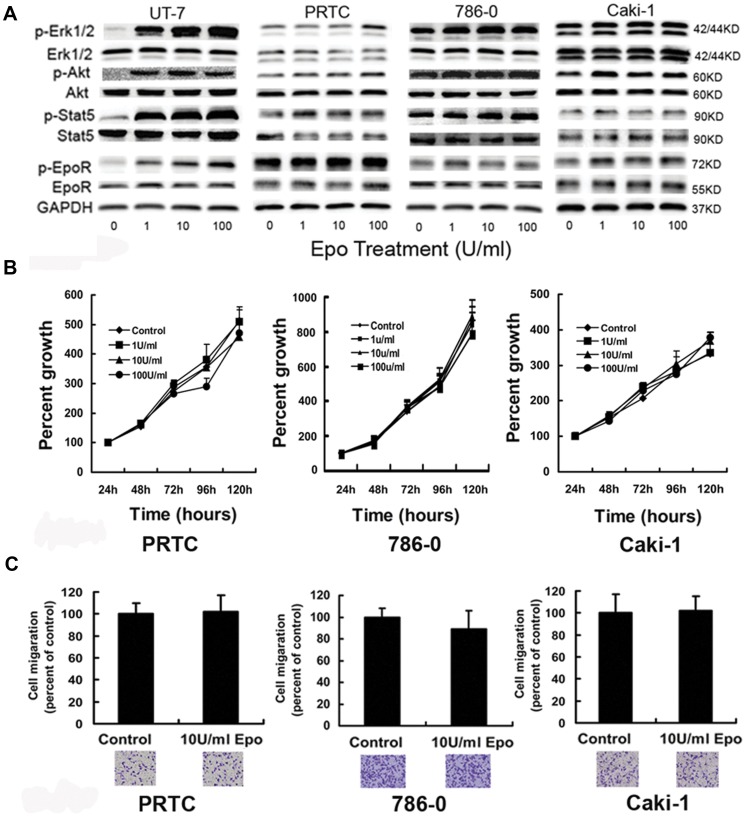
Exogenous Epo shows no biological effect on signaling molecules, proliferation, and invasion ability in RCC cells. (A) RCC cells were treated with 0–100 U/ml Epo for 5 min after serum starvation for 24 hours. Compared with Epo-dependent UT-7 cells, the phosphorylation of EpoR, STAT5, Akt and Erk1/2 in PRTC, 786-0 and Caki-1 cells increased insignificantly, if any, after rhEpo stimulations. Moreover, RCC cells expressed higher levels of baseline phosphorylation of all examined EpoR signaling proteins than UT-7 cells in the absence of exogenous Epo. In UT-7 cells, p-EpoR, p-STAT5, p-Erk1/2 and p-Akt were at lower levels before Epo stimulation, and increased significantly at 1 U/ml Epo. Experiments were done in triplicate with similar results. (B) PRTC, 786-0 cells and Caki-1 cells were cultured in media containing 0, 1, 10 and 100 U/ml Epo after serum starvation for 24 hours. Viable cells were evaluated after various incubation periods by modified MTT assay. Exogenous Epo had little effect on proliferation of RCC cells (6 wells for one sample, and experiments in triplicate; *P*>0.05 by one-way ANOVA). (C) Transwell tests for invasion ability of PRTC, 786-0 cells and Caki-1 cells. Upper chamber: 2×10^4^ RCC cells in serum-free medium with 0-100 U/ml concentrations of Epo; lower chamber: medium containing 10% FBS. Cells were stained and counted after incubation for 36 hours. Error bar represents mean ± SD (n = 3). There is no difference between RCC cells with Epo and those without Epo in medium (only showing 10 U/ml groups).

To assess the effect of exogenous Epo on Epo/EpoR pathway in RCC cells, we used the method similar to that described by Paragh et al. [25] The signaling molecules related to Epo/EpoR pathway were examined in RCC cells after treatment with various concentrations of Epo for a short period of time. Compared with rhEpo-dependent UT-7 cells, the phosphorylation of EpoR, STAT5, Akt and Erk1/2 in primary renal tumor cells, 786-0 and Caki-1 cells increased insignificantly, if any, after Epo stimulations. Moreover, RCC cells expressed higher levels of baseline phosphorylation of all examined EpoR signaling proteins than UT-7 cells in the absence of exogenous Epo (Figure 2A). In addition, RCC cells exhibited little or no response to exogenous Epo stimulation in cell proliferation by modified MTT test (Figure 2B), nor in cell invasion (*P*>0.05) by transwell test (Figure 2C).

### Signaling Molecules, Proliferation Rate, Apoptosis, and Invasiveness in 786-0 Cells with Knockdown EpoR

**Figure 3:**
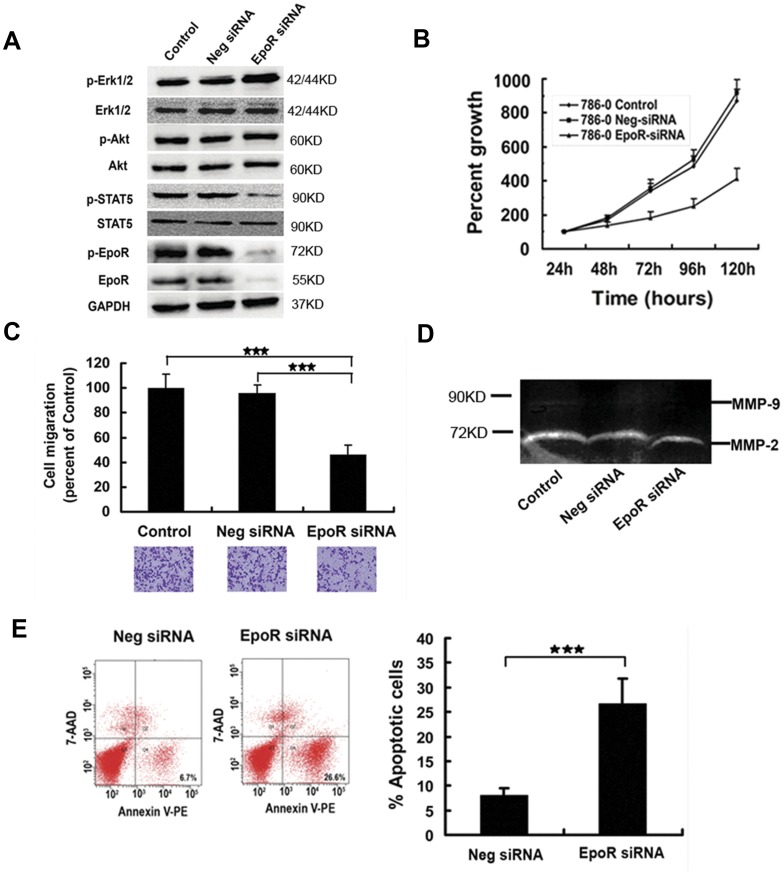
Change of signaling molecules, proliferation, apoptosis, invasion ability and MMP-2 expression in 786-0 cells with knockdown EpoR. (A) Signaling molecules were assayed in 786-0 expressing EpoR siRNA (EpoR siRNA) by western blot, using parental 786-0 cells (control) and 786-0 expressing negative siRNA (Neg siRNA) as controls. In 786-0 cells expressing EpoR siRNA, EpoR and p-EpoR decreased by >90%, p-STAT5 decreased by >60%, but p-Erk1/2 increased by >36%, as compared with those in the two controls. (B) Proliferation rate reduced significantly in 786-0 expressing EpoR siRNA by modified MTT method (experiments in triplicate; *P*<0.01), as compared with that at the same time point in the two controls. (C) Invasion ability decreased by about 55% in 786-0 expressing EpoR siRNA by transwell test (tests in triplicate; ★★★: *P*<0.001). (D) MMP-2 expression was inhibited by 33% in 786-0 expressing EpoR siRNA by gelatin zymography, and MMP-9 expression was very low in the 3 groups of 786-0 cells. (E) Early apoptotic cells (annexin V-PE^+^/7-AAD^-^) increased by about 4 times in 786-0 expressing EpoR siRNA, as compared to those of 786-0 expressing negative siRNA by flow cytometry (experiments in triplicate; ★★★: *P*<0.001).

In the cells stably expressing EpoR siRNA, EpoR and p-EpoR reduced by more than 90%, p-STAT5 decreased by more than 60%, whereas p-Erk1/2 increased by more than 36%, as compared with those in stably expressing negative siRNA and the parental cells (Figure 3A). 786-0 cells stably expressing EpoR siRNA showed slower cell proliferation rate by modified MTT test (Figure 3B), lower invasion ability (decreased by about 55%) by transwell test (Figure 3C), and lower MMP-2 expression (Figure 3D). In addition, 786-0 cells transiently expressing EpoR siRNA showed more apoptotic cells as compared with the two controls (Figure 3E).

### The Effects Epo/EpoR Pathway on Multi-kinases Inhibitor Sunitinib in 786-0 Cells

**Figure 4:**
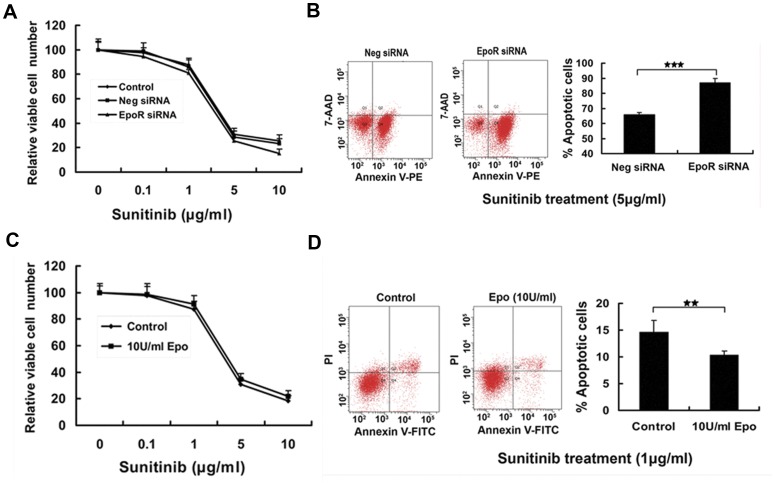
The effect of Epo/EpoR pathway on Sunitinib in 786-0 cells. (A) 786-0 cells stably expressing EpoR siRNA, 786-0 cells stably expressing negative siRNA, and parental 786-0 cells were incubated in the medium containing various concentrations of Sunitinib for 24 hours, and the viable cells were measured by modified MTT method. Error bar represents mean ± SD from 6 wells for each time point and triplicate experiments for each sample. 786-0 cells stably expressing EpoR siRNA were slightly more sensitive to Sunitinib than the two controls. (B) 786-0 cells stably expressing EpoR siRNA were cultured in medium containing 5 µg/ml Sunitinib for 24 hours, then subjected to assess cell apoptosis by flow cytometry. Suntinib treatment caused more apoptotic cells(Annexin V-PE^+^/7-AAD^+/−^)in 786-0 cells stably expressing EpoR siRNA than in 786-0 cells stably expressing negative siRNA (★★★: *P*<0.001). (C) 786-0 cells were exposed to various concentrations of Sunitinib with or without 10 U/ml Epo for 24 hours and assessed relative viable cell number by modified MTT assay. Error bar represents mean ± SD from 6 wells for each time point and triplicate experiments for each sample. Epo slightly antagonized the effect of Sunitinib. (D) 786-0 cells were cultured in medium containing 1 µg/ml Sunitinib with or without 10 U/ml Epo for 24 hours, and apoptotic cells (Annexin V-FITC^+^/7-AAD^+/−^)were measured by flow cytometry. Epo attenuated the effect of Sunitinib (*P*<0.01, from 4 repeated experiments).

Prior studies reported that exogenous Epo influenced the effects of chemoradiotherapy on cancer cells [11]. Recently, rhEpo were found to antagonize Trastuzmab (a monoclonal antibody against HER2) in breast cancer treatment [26]. Here we evaluated the role of Epo/EpoR pathway on the effects of Sunitinib. Sunitinib treatment induced slightly more apoptotic cells in 786-0 cells stably expressing EpoR siRNA than in the control cells examined by modified MTT method (Figure 4A) and flow cytometry (Figure 4B), indicating that 786-0 cells with knockdown EpoR became slightly more sensitive to Sunitinib. On the other hand, Sunitinib treatment induced less apoptotic cells in 786-0 cells in the presence Epo (Figure 4C and 4D), suggesting that the apoptosis effect of Sunitinb was partially antagonized by exogenous Epo treatment.

### Suppression of Xenograft Growth of 786-0 Cells with Knockdown EpoR

**Figure 5:**
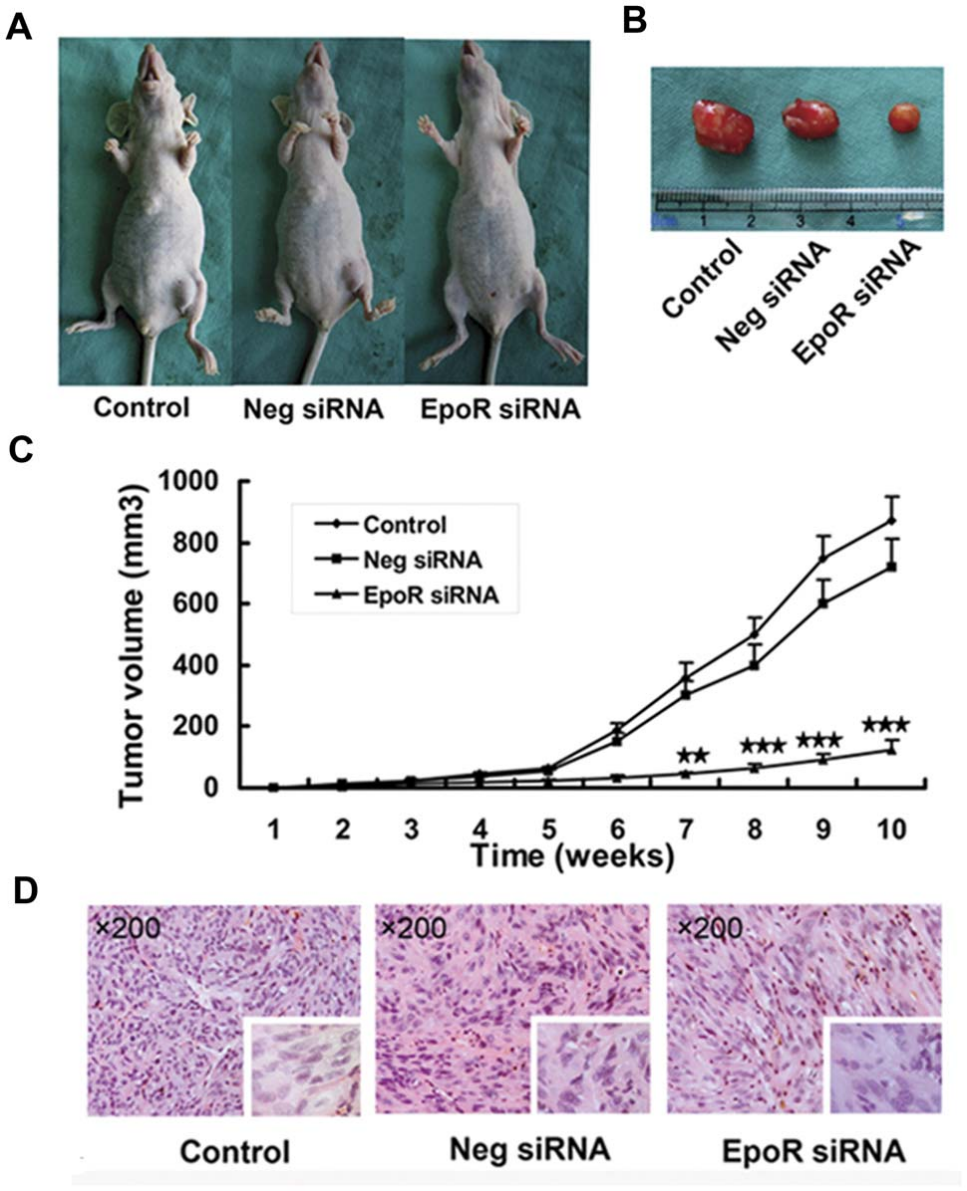
Knockdown EpoR suppresses 786-0 tumor growth in nude mice. Nude mice were subcutaneously inoculated with 2×10^6^ parental 786-0 cells (Control, n = 6), 786-0 cells stably expressing negative siRNA (Neg SiRNA, n = 7), or 786-0 cells stably expressing EpoR siRNA (EpoR siRNA, n = 10). (A) (B) Tumor sizes after the inoculation for 10 weeks. (C) Changes of tumor size within 10 weeks (★★: *P*<0.01, ★★★: *P*<0.001). (D) HE-stained xenograft slides showed the histology of clear cell RCC. Inserts ×400.

786-0 cells stably expressing EpoR siRNA were inoculated in nude mice, using 786-0 cell stably expressing negative siRNA and the parental 786-0 cells as controls. Tumor xenograft of 786-0 cells with knockdown EpoR grew significantly slower than the xenografts of the two control groups (Figure 5).

## Discussion

Epo and EpoR expression has been reported in many tumors including RCC, but the function of Epo/EpoR pathway is largely unknown in cancer cells. Subsequently, it has been a controversial issue whether rhEpo and its analog erythropoietin stimulating agents are detrimental for the treatment of anemia in cancer patients by promoting cancer cell survival and angiogenesis [10]–[11], [27]–[29]. In this study, we used cultured human primary renal cancer cells and RCC cell lines including 786-0 (VHL-mutant) and Caki-1(VHL-normal) to study the role of Epo/EpoR pathway on cell proliferation, apoptosis, invasiveness, and sensitivity to anti-tumor drug Sunitinib in RCC cells. We used exogenous Epo to stimulate Epo/EpoR pathway in RCC cells. We also down-regulated EpoR expression by lentivirus-introduced siRNA method, from which a 786-0 cell line with stably down-regulated EpoR was established. Our findings suggested that Epo/EpoR pathway was involved in, at least partially, promoting cell proliferation and invasiveness, and reducing cell apoptosis and sensitivity to anti-tumor drug Sunitinib in RCC. Therefore, EpoR may be considered as a candidate of anti-RCC target for further study.

Using quantitative real-time reverse-transcription-PCR (qRT-PCR) and western blot, we found that primary renal cancer cells and the 6 RCC cell lines including 786-0, 769P, A498, OS-RC-2, Caki-1 and Caki-2 cells expressed EpoR and Epo, consistent with the findings from human RCC surgical samples [12], [17] and suggesting the existence of autocrine activation of EpoR pathway in RCC. Interestingly, primary renal cancer cells, 786-0 and Caki-1 cells were unresponsive to exogenous Epo, and cells cultured in 1–100 U/ml Epo showed the growth rate and invasion ability similar to those of negative controls. Phosphorylations of the EpoR-related signaling molecules were at higher levels even in the absence of exogenous Epo and after serum starvation for 24 hours, and exogenous Epo had little or no further increase of phosphorylation levels, indicating the constitutional activation of Epo/EpoR signaling pathway in primary renal cancer cells, 786-0 and Caki-1 cells. The mechanism underlying this phenomenon is currently unknown. Similarly, increased baseline phosphorylation of EpoR signaling proteins and no biological effects of exogenous Epo were also found in A2780 human ovarian carcinoma cell line. In A2780 cells, the lack of Epo expression excluded the possibility of autocrine activation, and the cause of fully activated EpoR signaling pathway at baseline condition was unclear [25]. The authors proposed that there may be an Epo-independent, EpoR-mediated pathway in the growth of some human cancers. In another previous study, 786-0 cells were found neither to express Epo mRNA nor secrete Epo, and Epo stimulation could induce cell proliferation [10]. The differences may result from the methods used in the experiments. Our results seem convincing, because we used qRT-PCR to measure Epo mRNA expression, and we observed the effect of Epo on cell proliferation for 5 consecutive days.

To evaluate the function of EpoR in RCC cells, we used RNA interference method to down-regulate EpoR in 786-0 cells, and examined the changes of signaling molecules, proliferation, invasion, apoptosis, xenograft growth *in vivo*, and sensitivity to RCC targeted drug Sunitinib. Compared with the parental 786-0 cells and the cells transfected with scrambled siRNA cells, 786-0 cells with down-regulated EpoR exhibited retarded tumor growth *in vitro* and *in vivo*, increased cell apoptosis, and decreased invasion ability demonstrated by the reduced cell migration and MMP-2 expression. EpoR is a member of the cytokine receptor superfamily. Epo binding to EpoR induces the phosphorylation of EpoR, JAk2 and STAT5, and STAT5-responsive genes are consequently induced [30]. EpoR activation also induces the activation of MAPK and AKT pathways that relate to the mitotic and anti-apoptotic signals of Epo [30]–[31]. As expected, p-EpoR and p-STAT5 were significantly decreased in 786-0 cells with down-regulated EpoR. However, p-AKT remained unchanged, and p-Erk1/2 increased. Similar findings were found in ovarian carcinoma A2780 cell with down-regulated EpoR, in which p-AKT increased for compensatory mechanisms [25]. In melanoma cells, EpoR down-regulation resulted in diminished p-Erk in response to Epo stimulation [29]. Further investigations are needed to disclose other functions of EpoR in RCC cells.

Sunitinib is a multi-targeted receptor tryrosine kinases inhibitor effective for the treatment of RCC. However, this drug only extends patients’ life, drug resistance usually occurs after a median of 6–15 months of treatment [32]. To explore whether Epo/EpoR signaling pathway is involved in the resistance to Sunitinib through its promotion of cell proliferation and inhibition of apoptosis, we compared the killing effect of Sunitinib on parental 786-0 cells and the cells with down-regulated EpoR in the presence and absence of exogenous Epo. The sensitivity to Sunitinib was slightly reduced by exogenous Epo in 786-0 cells. As Epo/EpoR pathway is constitutively activated in 786-0 cells, the result may reflect the fact that Sunitinib partially inhibits the phosphorylation of EpoR. In addition, the sensitivity to Sunitinib in 786-0 cells was slightly enhanced by down-regulation of EpoR in the cells, suggesting that Epo/EpoR pathway may play a partial role in the resistance to Sunitinib.

In conclusion, co-expression of Epo and EpoR was found in RCC cells. EpoR and its signaling molecules STAT5, Akt, and Erk1/2 were constitutively activated in primary renal cancer cells, 786-0 cells and Caki-1 cells. Exogenous Epo had no additional effects on proliferation and invasion ability of the cells. Down-regulation of EpoR expression in 786-0 cells attenuated the proliferation and invasion *in vitro* and *in vivo*, and the resistance to Sunitinib. Exogenous Epo slightly antagonized the effects of Sunitinib on 786-0 cells. We propose that Epo/EpoR signaling pathway may be functional, and inhibition of the pathway may result in the decrease of cell growth, migration, survival, and resistance to Sunitinib in RCC cells.

## Materials and Methods

### Cell Culture

The cell lines used in this study included six human RCC cell lines (786-0, 769P, A498, OS-RC-2, Caki-1 and Caki-2). HK-2 (a normal human proximal tubular cell line), HepG-2 (a human hepatoma cell line expressing Epo), and UT-7 cells (an Epo-dependent human leukemia cell line) were used as the control cell lines. 786-0 cells, a human renal cell line carrying a mutation in *VHL* gene and representative of the most frequently encountered renal carcinomas, were purchased from ATCC (Rockville, MD, USA). Other cell lines including 769P, A498, OS-RC-2, Caki-1 (without VHL mutation), Caki-2, HK-2, HepG-2 and UT-7 were obtained from Chinese Academy of Medical Science & Peking Union Medical College Cell Culture Center (Beijing, China). 786-0, 769P, OS-RC-2 and HepG-2 were maintained in RPMI 1640 medium, A498 in MEM, Caki-1 and Caki-2 in MoCoy’s 5a medium. HK-2 cells were grown in a 1∶1 mixture of DMEM/F12. UT-7 cells were cultured in RPMI 1640 medium with the supplement of 7 U/ml rhEpo (Epogen, Amgen, Thousand Oaks, CA, USA). All media contained 10% fetal bovine serum (FBS) (GIBCO Invitrogen, Carlsbad, CA, USA). To test the short-term effect of rhEpo on cells, sub-confluent cultures of primary renal tumor cells, 786-0, Caki-1and UT-7 cells were treated with rhEpo for 5 minutes in six-well plates after FBS and Epo starvation for 24 hours.

### Culture of Primary Renal Tumor Cells

Samples of RCC were obtained from a patient who underwent nephrectomy in Peking University First Hospital. The renal tumor block was cut into small pieces, digested with collagenase for 4 hours, and then with trypsin for 1 hour. The cells were washed twice in RPMI 1640, and then cultured at 37°C in an atmosphere of 5% CO2. Cells were retrieved by trypsinization and passage when they reached a confluence of 90–95%. Cells were collected at 2nd passage. The protocol for the primary renal tumor cell culture was approved by the Medical Ethics Committee of Peking University First Hospital.

### Lentivirus RNA Interference Construct and Transfection

We designed oligonucleotides targeting human EpoR mRNA of 5′-AACTACAGCTTCTCCTACCAG following the sequence used by Paragh et al [25], and the scrambled negative control sequence of 5′-TTCTCCGAACGTGTCACGT not complementary to any known human cDNAs, cloned the designed oligonucleotides into lentivirus vector between U6 promoter and green fluorescent protein (GFP) cDNA, sequenced the vectors to confirm the inserts, and then transfected the packing cells for the generation of recombinant lentivirus. 786-0 cells were plated in six-well plates at a density of 2×10^5^ cells per well, incubated overnight, and transfected with 4×10^6^ recombinant lentivirus for 8 hours. Effective introduction of EpoR siRNA into 786-0 cells was confirmed by the presence of green fluorescence in cytoplasm under a fluorescence microscope. For generation of a stably knockdown EpoR cell line, cells after transfection for 48 hours were trypsinized and plated at a ratio of 1∶10 for several times. Cells with GFP fluorescence indicating the stable expression of EpoR siRNA were cloned and confirmed by the lower EpoR expression on western blot.

### Quantitative Real-time PCR Assay

Total RNA was extracted from cells using Trizol reagent (Invitrogen, Carlsbad, USA). Reverse-transcription (RT) was performed using 2 µg total RNA, oligo (dT) primer and AMV reverse transcriptase in a volume of 20 µl. Quantitative RT-PCR was carried out in a 7300 real-time PCR system (ABI, Foster City, USA) using TaqMan probes as the indicator and the default condition set in the instrument. For measurement of Epo cDNA, we used primer Epo-F: 5′-GCAGCCTCACCACTCTGCTT, primer Epo-R: 5′-CGGAAAGTGTCAGCAGTGATTG, and Epo TaqMan probe: 5′-FAM-TCTCCCCTCCAGATGCGGCCTC-TAMRA. For measurement of EpoR cDNA, we used primer EpoR-F: 5′-AGCCCAGAGAGCGAGTTTGA, primer EpoR-R: 5′-CCACAGGCAGCCATCATTCT, and EpoR TaqMan probe: 5′-FAM-TCACCACCCACAAGGGTAACTTCCAGCT-TAMRA. We also measured GAPDH cDNA in the samples as the loading reference, using primer GAPDH-F: 5′-CAGTCAGCCGCATCTTC- TTTT, primer GAPDH-R: 5′-GTGACCAGGCGCCCAATAC, and GAPDH TaqMan probe: 5′-FAM-CGTCGCC-.

AGCCGAGCCACA-TAMRA.

### Human Epo ELISA Assay

When RCC cells grew to confluence, the medium was used for Epo determination. We used the Human Epo Elisa Assay kit (RB, CA, USA) following the manufacturer’s recommendation. Each sample was performed in triplicate.

### Western Blotting

Cells were washed twice with ice cold phosphate-buffered saline (PBS) and lysed in RIPA buffer (50 mmol/l Tris-HCl pH 7.4, 150 mmol/l NaCl, 1 mmol/l EDTA, 0.25% sodium deoxycholate, 1% NP-40, 0.1 mg/ml PMSF, 10 µg/ml aprotinin, and 1 mmol/l sodium orthovanadate). Lysate containing 40 µg protein quantified by BCA Protein Assay Kit (keyGEN, Nanjing, China) for each sample was subjected to SDS-PAGE, transferred to nitrocellulose membrane, and blotted by the primary antibodies against EpoR (M-20, sc-697, Santa Cruz, CA, USA), phosphorylated-EpoR (p-EpoR) (rabbit polyclonal antibodies, Santa Cruz, Santa Cruz, CA; 1∶500), STAT5, p-STAT5, Akt (PKB), p-Akt, Erk1/2 or p-Erk1/2 (rabbit polyclonal antibodies, Cell Signaling, Danvers, MA; 1∶1000), or GAPDH (mouse monoclonal antibody, Santa Cruz, CA, USA) as a loading reference. Blotted primary antibodies were detected by 1∶5000 HRP-conjugated anti-rabbit or anti-mouse IgG secondary antibody and enhanced chemiluminescence (ECL), and visualized by Imaging Station 4000 mm Pro (Kodak).

### Cell Proliferation Assay

Viable cells were assessed using a modified MTT assay kit (keyGEN, Nanjing, China) following the manufacturer’s protocol. To determine the stimulating effect of rhEpo on cell proliferation, RCC cells were plated in 96-well plates at a density of 1,000 cells per well, incubated to adhesion, cultured in serum-free medium for 24 hours, and then cultured in medium containing 10% FBS and 0 to 100 U/ml rhEpo. The amount of viable cells was determined every 24 hours using six wells per time point. For color development, 10 µl dye solution was added to each well and the plate was incubated at 37°C for 3 hours. Absorbance at 450 nm was determined using a 96-well plate reader. Each sample was performed in triplicate.

### Flow Cytometry

To assay the apoptosis effect of down-regulated EpoR, 786-0 cells were transiently transfected with the recombinant lentivirus vector for 3 days for down-regulation of EpoR mRNA, then gently trypsinized and washed with PBS, re-suspended in 50 µl 1× binding buffer (10 mmol/l HEPES buffer pH 7.4, 150 mmol/l NaCl, 1 mmol/l CaCl_2_), and stained with PE-conjugated annexin V and 7-Aminoactinomycin D (7-AAD). To determine the anti-apoptosis effect of rhEpo, 786-0 cells were plated in six-well plates at a density of 5×10^5^/well, cultured to sub-confluence, treated with Sunitinib with or without rhEpo in the medium for 24 hours, trypsinized and washed twice with PBS, re-suspended in 50 µl 1 × binding buffer, and stained with FITC-conjugated annexin V and propidium iodide (PI). Cells were then incubated at room temperature for 10 minutes in dark, analyzed in a flow cytometer (FACSAria, BD) within one hour. Each sample was done at least in triplicate.

### Cell Invasion Assay

The 24-well Biocoat Matrigel Invasion Chamber with an 8 µm pore polycarbonate filter (Becton Dickinson, Bedford, MA, USA) was used for the assay. Cells in a growing phase were trypsinized, re-suspended in serum-free medium with or without Epo, and plated in upper chamber at a density of 2×10^4^ cells. The lower compartment was filled with medium containing 10% FBS. After incubation for 36 hours, cells on filter were stained and counted under a microscope.

### Gelatin Zymography Assay

Matrix metallopeptidase (MMP) activities in 786-0 cells were determined by gelatin zymography. The sample extracted from cell supernatant was diluted in a buffer (0.12 M Tris–HCl, 20% glycerol, 0.1% bromophenol blue, 10% SDS), and 10 mg total protein was separated in gelatin-impregnated zymogram gel (10% zymogram gelatin gel) at 120 V for 90 min. The gel was incubated at room temperature in a zymogram renaturing buffer (Invitrogen, CA) for 30 min, washed in zymogram developing buffer for 30 min, incubated in fresh zymogram developing buffer overnight at 37°C, developed by staining with 0.5% Coomassie blue for 90 min, and washed in destaining solution until clear bands of MMPs appeared against a blue background.

### Tumourigenesis Assay in Nude Mice

Thirty BALB/C nude mice were randomly divided into three groups and then subcutaneously injected into left groin area with 2×10^6^ 786-0 cells stably expressing EpoR siRNA, 786-0 cell expressing scrambled negative siRNA and parental 786-0 cells. Tumor size was recorded every week for 10 weeks. At the end point, 6, 7 and 10 nude mice remained in parental cell group, negative siRNA group, and EpoR siRNA group, respectively. The animal experiment protocol was approved by Peking University Institutional Animal Care and Use Committee.

### Statistical Analysis

Data are expressed as mean ± SD. Comparisons among groups were conducted by one-way ANOVA followed by the least square difference test. Paired *t*-test was used for comparison between two groups. *P*<0.05 was considered to be significant.
